# Supplementary material for: Patterns of Intron Gain and Loss in Fungi
Source: PLoS Biol. 2004 Nov 30;2(12):e422. doi: 10.1371/journal.pbio.0020422 (PMC532390; doi:10.1371/journal.pbio.0020422)
Supplement: Table S1 — Also available at http://genes.mit.edu/NielsenEtAl/. (4.3 MB ZIP). [file pbio.0020422.st001.zip › NielsenEtAl/html/344.html]

AN3150.1.NCU01157.1.MG07317.1.FG09991.1


```
 CLUSTAL W (1.82) Multiple Sequence Alignments - Introns Inserted


Sequence 1: MG07317.1	695 aa
Sequence 2: FG09991.1	717 aa
Sequence 3: NCU01157.1	728 aa
Sequence 4: AN3150.1	621 aa
Alignment Length: 751 aa
Number Identitical Residues: 378 aa
Alignment Score (without introns) 18121


MG07317.1 	MGLL2ALGTALEWPEAKKHAHQVREWGIK0QLLEIWNKAKHKERDALLWGDE0V------
NCU01157.1	MGLL2ALGTALDWPDAKQQAHLVRAWGIK0QLLEIWNKAKGKERDAMLWGDE0I------
FG09991.1 	MGLL2ALGTALDWPDAKKRAPQVREWGIK0QLLEIWNKAKGKERDALLWGDE~V------
AN3150.1  	MGLL2ALGTALEWPEAKKRAD-------Q0QLLANWHRAKGKERDALLWGDE~VCALFVA
          	**** ******:**:**::*        : ***  *::** *****:***** :.:   :

MG07317.1 	~-EYLVVVFDQDDPNVKLSLRQAEILEA~LAASPDLSAKGGCVPALQDTSATE2E-S~EE
NCU01157.1	~-EYLVVNYSKDEPKVLLSLRQADILKA~LADSKTLKVTGDCAPGTEANADAG~N--~-V
FG09991.1 	~-EYLVVVYSEDNKRVLLSLRQAQILEA~LAADKDLKKQGGCVPELQEPTTTE~KKT2ER
AN3150.1  	0VEYLVVALDDAAKKARLSLAQADILKS2EGEEP-------------------~---~--
          	  *****  ..   .. *** **:**::  . .                           

MG07317.1 	PLPVFHPEFGRFMLEATPGKPWGIGFKELLDVEPNMKLR~RTIAKQHMRADEYPITLTTY
NCU01157.1	TLPVFHPEFGRFMLEATPGKPWGIDFKDLLTVEADMKLR~RCIAKDHMLSNEHPITLTTF
FG09991.1 	TIPVFHPEFGRFMLEATPGKPWGIGFKELLDVEPDMKLR~RKIAKEHMLSTEYPITLTTY
AN3150.1  	--PHFHPEFGRFMLEATPGVPWGIGFKDLLKVESNMRWR2REVAKAHMAPNESPITLTTF
          	  * *************** ****.**:** **.:*: * * :** ** . * ******:

MG07317.1 	PRIGCPEKFTDPYYPPSGPKLRSQFVPDEIANPHIRFPTLAANIRSRRGRKVQVNVPVFR
NCU01157.1	PRIGSPGVFTEPSYPVSGPKLRSQFVPDEIANPHIRFPTLAANIRSRRGRKVQVNVPVFR
FG09991.1 	PQIGVPGQFTDPYFPPSGPRLRSQFVPDEIANPHIRFPTLAANIRSRRGRKVQVNVPIFH
AN3150.1  	PRLGTKDDYIQPYYPPSGPALRSQFVPDEIANPHIRFPTLAANIRARRGRKVELNVPVFK
          	*::*    : :* :* *** *************************:******::***:*:

MG07317.1 	DEKTPWPWTDPTVNRDMHNWPEDEDVRSGGAAPDNFIHMDAMAFGMGSCCLQITFQAKNI
NCU01157.1	DENTPWPWKDPTVNYDLHNWPEDDDVRNG-AAPDNFIHMDAMAFGMGSCCLQITFQAKNI
FG09991.1 	DKNTPKPWKDPTVNLAKYDWPEDDDVRNG-AAPDDFIHMDAMAFGMGSCCLQITFQAKNI
AN3150.1  	DKNTPEPFKDPTVNYDLHNWPEDDDVRNG-AAKDGHVYMDAMAFGMGSCCLQITFQAKNV
          	*::** *:.*****   ::****:***.* ** *..::*********************:

MG07317.1 	IEGRTMYDQLSPLGPILLALTAATPIYKGFLADTDVRWNQISRAVDCRTKEEMGEE0PLK
NCU01157.1	TEGRKMYDQLSPLGPILLALTAATPVYKGFIADTDVRWNQISRAVDDRTPEELGEK0PLK
FG09991.1 	TEGRMLYDQLSPLGPIMLALTAATPVYKGFVANTDVRWNQISRAVDCRTPEELGEK0PLT
AN3150.1  	PEGRKLYDQLSPLGPILLALTAATPIYKGFLVDTDVRWNQISKAVDDRTREELG--~---
          	 *** :**********:********:****:.:*********:*** ** **:*      

MG07317.1 	NDRWRIPKSRYASNSTYIST~DHRLRPEYMDPNLVIDEDIKAKLVEGGMDDLLATHFAHL
NCU01157.1	NDRWRIPKSRYASNSTYIST~DSRLRPEYMDPNLVIDPEIKQQLLDGGMDDRLATHFAHL
FG09991.1 	EGVRRIPKSRYASNSTYIAI~DPRLRNEYLDPDLVYDSDIKRQLLEGGMDDRLATHFAHL
AN3150.1  	------------------EV0DPRLRKEYLDPDLIVDEDIKKRLMDDGMDELLATHFAHL
          	                     * *** **:**:*: * :** :*::.***: ********

MG07317.1 	FIRDPIVIFAEDLERLDLEKTDHFENLQSTNWQHMRFKPPPADN-NIGWRVEFRPMEIQV
NCU01157.1	FIRDPIVIFNEDLQELDLTKTDHFENIQSTNWQHMRFKPPPADN-SIGWRVEFRPMEIQI
FG09991.1 	FIRDPIVVFEEDLQELDLNKTDHFENIQSTNWQHMRFKPPPADN-SIGWRVEFRSMEIQI
AN3150.1  	FIRDPIVIFSEDLEELDLNKADHFENLQSTNWQHMRFKPPPPEKDDIGWRVEFRSMEIQM
          	*******:* ***:.*** *:*****:**************.::..********.****:

MG07317.1 	TDFENAAFSVFIVLVTRAILSFNLNFYIPIQRVDENMETAHARDAVLEKKFWFRRNLFPT
NCU01157.1	TDFENAAFSVFIVLITRAILSFDLNFYIPIVKVDENMETAHARNANLEKKFWFRKNPFPV
FG09991.1 	TDFENAAFSVFMVLVTRAILSFDLNFYIPIKKVDENMERAHEVDAVLKEKFYFRRNPFPS
AN3150.1  	TDFENAAFSIFIVLVTRAILSFDLNFYIPIQRTAENMETAHARNAVLDRKFYFRKDPFSP
          	*********:*:**:*******:******* :. **** **  :* *..**:**:: *. 

MG07317.1 	RSPR-----GGGSASGTATPF---SRSPTP-VGPVDDEYRLMTINEIINGTTAAAGSTHD
NCU01157.1	RTTRRGGS-ASRSASGTSTPNSGSSRPATPPLGPVEDEYRLMSVNEVINGTAYAKATSKE
FG09991.1 	RPSRANTTFGDDSRPGSAHP----SRPPSP-GGPVEHEFEEMTVNDIINGSESGE-----
AN3150.1  	SVRRHHNS-SGDSNTSSANN----TPPPSPPLGPVEYEFELMTISDIINGSADGS-----
          	   *   : .. * ..::      : ..:*. ***: *:. *::.::***:  .      

MG07317.1 	DHDDANDDDHFPGLIPIVESYLDSVNVDVETRCHLATYLDLIRRRASGELSTAARWIRDF
NCU01157.1	VTEDAEEGEEFPGLIPLVESYLDSVNMDVATRCGLARYLDLIRKRASGELWTAAKWIREF
FG09991.1 	----------FPGLIPIVESYLDSVNVDVSTRCHLSTYLDLISKRARGDLDTTARWIRNF
AN3150.1  	----------FPGLIPLVESYLNSVNVDVETRCSLARYLDLIRKRANGTLWTGAKWIREF
          	          ******:*****:***:** *** *: ***** :** * * * *:***:*

MG07317.1 	VAAHPKYEKDSVVGPEINHDLVKAIIAIGEKECAGKGFKGID--VFGFEKLLGNFRGG--
NCU01157.1	IAKHPGYKKDSVVSEEITKDLVGAVIEIGEREKRGLGIDDLIGQVPDLEKLFGGFLKGKS
FG09991.1 	IDIHPKYNHDSVVNDEINHDLIGAVIAIGERETAGRNFAGLG--IHGLERLLNGFRGG--
AN3150.1  	VAQHPAYKQDSVVSEEICYDLVKAVEEMSVKEGADG--------SVGWEMLKGRTV----
          	:  ** *::****. **  **: *:  :. :*  .           . * * .       

MG07317.1 	-CGGPQSS---------------------------------
NCU01157.1	PCGGGQAALEQKLK--DDDAKVNGVKRKFNEEQ--------
FG09991.1 	-CGGGSSNGSNGNSHAAETETVNGSLKRKSEWIDGQEVMAS
AN3150.1  	-----------------------------------------
          	
```
